# Cardiac organoids: tailoring presurgical drug testing for heart failure patients

**DOI:** 10.1097/MS9.0000000000004556

**Published:** 2025-12-11

**Authors:** Syeda Rabiah Shahid, Muhammad Talha, Araj Naveed Siddiqui, Hermann Yokolo

**Affiliations:** a Department of Internal Medicine, Shaikh Khalifa Bin Zayed Al-Nahyan Medical and Dental College, Lahore, Pakistan; b Department of Internal Medicine, King Edward Medical University, Lahore, Pakistan; c Department of Internal Medicine, Jinnah Sindh Medical University, Karachi, Pakistan; d Department of Research, Medical Research Circle (MedReC), Goma, DR Congo

*Dear editor,*

Cardiovascular disease and resultant heart failure are a major cause of morbidity and mortality, with a global prevalence of over 64 million people^[1]^. While pharmacotherapy can reduce the progression and burden, the ultimate treatment for heart failure remains surgical transplant or a more novel surgical approach to provide mechanical circulatory support^[2]^. However, postsurgical outcomes and treatment plans remain ambiguous owing to the vulnerable condition of the patient, heterogeneity of the disease itself, and variable responses even to the standard treatment^[3]^. This article aims to promote the use of cardiac organoids for personalized pharmacological tests before surgery in patients with heart failure. This article complies with the TITAN 2025 guidelines – repressing the reporting and use of AI^[4]^.

Cardiac organoids are lab-grown cardiomyocytes derived from human induced pluripotent stem cells (iPSCs) developed into three-dimensional (3D) structures, extensively employed in modeling cardiac disease and the development of drugs in vitro studies^[5,6]^. These are now emerging as a tangible and reliable clinical tool. As medicine evolves towards a more personalized and targeted treatment paradigm, the use of cardiac organoids in presurgical drug testing for heart failure patients becomes imperative in enhancing surgical outcomes and refining therapy. These 3D models can mimic cellular heterogeneity, contractile function, electrophysiological properties, and potential vascular and chamber-specific architecture^[5]^. Moreover, recent breakthroughs in vascularization of the 3D structure with branching capillaries further underscore the potential it holds, thereby offering a tentative framework for personalized pharmacologic optimization^[7]^.

By exposing these lab-grown heart models to various inotropes, vasodilators, and neurohormonal modulators before surgery, a unique, patient-specific contractile and electrical response can be mapped out-tailoring dosing regimens^[5]^. Actionable pharmacologic metrics could include contractility thresholds (e.g., fractional shortening >20% under inotrope challenge), QT interval prolongation (>450 ms indicating arrhythmogenic risk), and vascular reactivity (e.g., >30% change in capillary diameter to vasodilators), directly informing perioperative dosing and safety thresholds. Concomitantly, these organoids reflecting the electrophysiological and vascular properties of diseased myocardium enable early detection of dangerous effects, including but not limited to QT prolongation and unfortunate vasoreactivity. Moreover, functional efficacy and burden tolerability of adjunctive therapy with diuretics and volume-minimizing agents, as is common in heart failure patients, can be gauged before initiating them in the patient. In patients with comorbid conditions and high susceptibility to polypharmacy-linked drug interactions, hybrid organoid models can identify non-specific secondary toxicities, thus providing unprecedented foresight^[5,7]^. Patients undergoing advanced cardiac surgery can benefit from presurgical drug testing on these powerful clinical tools by precisely optimized hemodynamic therapy, assessing arrhythmogenicity or vascular reactivity, and postoperative adjunctive pharmacotherapy to optimize safety and efficacy in contrast to traditional perioperative approaches^[8]^.

A major practical barrier to implementing patient-specific organoids for presurgical testing is the time required for iPSC derivation and organoid maturation, which typically spans weeks to months and may conflict with urgent surgical timelines^[5]^. Potential solutions include banking pre-derived iPSC lines from high-risk heart failure cohorts in advance, accelerating differentiation via high-throughput protocols (e.g., small-molecule modulation)^[5,9]^, or using allogeneic “off-the-shelf” organoids matched for key genetic variants predictive of drug response, with validation in nonurgent elective cases to build predictive datasets^[5]^.

Despite the potential advantages of using organoids to personalize heart failure treatment, certain challenges persist. Limited adult cardiomyocyte models, left and right chamber asymmetry, and lack of multicellular composition, including the accessory, supporting cells, warrant further advancements in the development of improved prototypes^[5]^. Current maturation techniques, such as electrical stimulation, metabolic conditioning with fatty acids, and coculture with endothelial or fibroblast populations, are progressively overcoming fetal-like phenotypes to better recapitulate adult myocardium^[5,9]^. These limitations can be ameliorated by adopting techniques that mature organoids to reflect adult myocardium, establish reproducible metrics for contractility and electrical activity, minimize ischemic necrosis by enhanced vascularization within 3D models, and validate organoid predictions against surgical outcomes^[7,9]^. Predictive accuracy could be benchmarked through prospective pilot trials correlating organoid responses with postoperative hemodynamics, arrhythmia incidence, or drug tolerability, or via retrospective analyses linking in vitro metrics to electronic health record outcomes. Collaborative efforts between stem cell biologists, anesthesiologists, and cardiothoracic surgeons are essential to transform a futuristic conceptual potential into a real-time clinical routine. Furthermore, pilot studies underscoring the potential outcomes by integrating such models can further expand the use and identify potential areas for improvement.

In the era of personalized medicine, cardiac organoids represent not only an in vitro laboratory tool but a potential opportunity for precision cardiac surgery. By harnessing such techniques for presurgical drug testing on an already vulnerable patient population, heart failure management can truly be revolutionized-placing the patient’s biology, quite literally, at the heart of the treatment plan. Despite the limitations, this innovative approach carries immense potential for integration into clinical practice, offering a paradigm shift from traditional practices to a personalized, patient-specific approach.

## Data Availability

Not applicable.
